# Stemming techniques for resource-poor languages: a review of methods, challenges, and applications

**DOI:** 10.3389/frai.2026.1875268

**Published:** 2026-09-22

**Authors:** Sanjiban Sekhar Roy, Mohd Anas, Saravanakumar Kandasamy

**Affiliations:** School of Computer Science and Engineering, Vellore Institute of Technology, Vellore, Tamil Nadu, India

**Keywords:** deep learning, morphologically rich languages, natural language processing, resource-poor languages, stemming techniques

## Abstract

The process of stemming is a fundamental part of pre-processing in NLP systems in resource-poor and morphologically complex languages in which linguistic data and annotated corpora are limited. This survey presents a a systematic review of stemming techniques across Afro-Asiatic, Indo-Aryan, Turkic, and Uralic language families, with explicit acknowledgment of coverage limitation and it covers rule-based, statistical, unsupervised, hybrid, and emerging neural-assisted approaches. The evolution of stemming is analyzed in the broader context of NLP paradigms, from early suffix-stripping algorithms to modern sub-word-aware and transformer-influenced models. Special emphasis has been given on resource-poor languages such as Arabic, Urdu, Bengali, Assamese, and other low-resource Indo-Aryan, Afro-Asiatic, and agglutinative languages. For these languages performing explicit stemming remains a critical task despite advances in deep learning. This study further reviews application-driven impacts of stemming in information retrieval, text classification, sentiment analysis, topic modeling, and semantic similarity estimation. A unified comparison framework is presented which has incorporated benchmark datasets, shared-task resources, and multi-dimensional evaluation metrics, including precision, recall, F-measure, MAP, NMI, and stemming-specific error indices. Using careful comparative evaluation, this study points out the strengths and weaknesses of current approaches and provides insights into potential areas for future investigation and developments in language-sensitive stemming systems.

## 1 Introduction

Stemming is a crucial tool used in natural language processing (NLP) to perform normalization by reducing words to their root form (Nathani et al., 2020). Stemming helps in improving the efficiency and accuracy of text processing algorithms. Stemming unifies different word forms under one root, improving text comparison and analysis. The earliest algorithmic model for stemming was introduced in 1968 by Julie Beth Lovins, Massachusetts Institute of Technology (Lovins, 1968). The most widely known and influential techniques for stemming is the algorithm of “Porter stemming.” This was was propsoed in 1979 by Martin Porter Porter Stemmer is a rule-based algorithm (Porter, 1980). The below Figure 1 shows the process of the generic NLP pipeline for stemming, spanning input sources, preprocessing, stemming paradigms, modeling, evaluation, and downstream applications. Over the past few decades, interest and enthusiasm for developing and exploring techniques to manage textual data have steadily increased (Ali et al., 2017). AS it helps in tasks such as categorization system, text summarization, text classification, sentiment analysis such as hate speech detection (Rajalakshmi et al., 2023), search engines, and information retrieval (Jaafar et al., 2017; Göksel et al., 2023). Stemming can also help in reducing the size of text data by removing redundant word variations, which can improve the performance of NLP algorithms. However, stemming is not always perfect and can sometimes result in the loss of meaning or introduce errors, which must be carefully considered when using stemming techniques.

**Figure 1:**
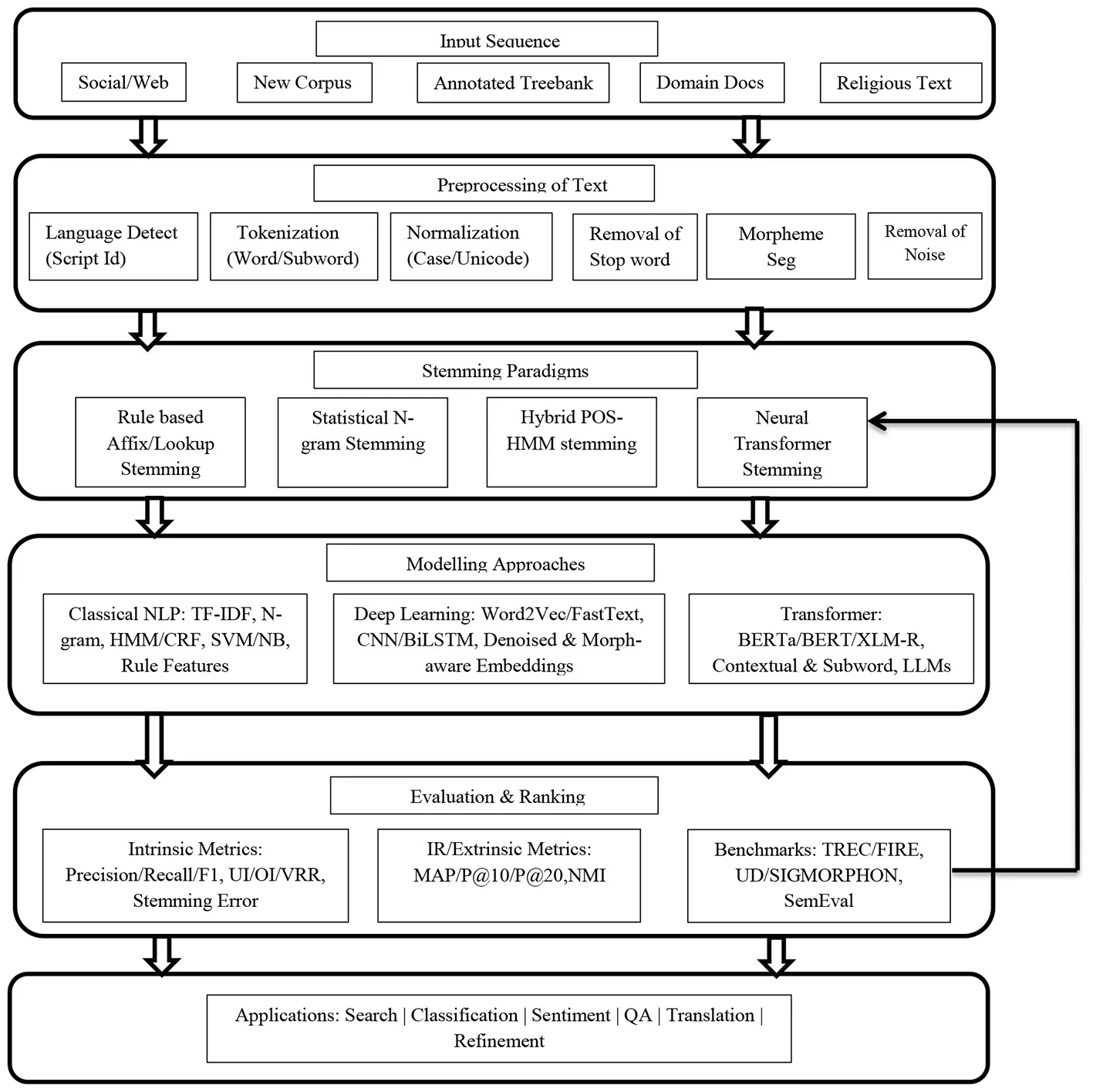
Efficient NLP pipeline for stemming in low-resource languages.

Lemmatization is an alternative technique applied in NLP for preprocessing purposes. While stemming removes affixes to obtain a root form, lemmatization uses morphological analysis and dictionary lookup to determine the base or dictionary form of a word Straková et al. (2014). Lemmatization can handle irregular forms: for instance, applying stemming to the word “better” can yield a truncated form like “bett,” whereas lemmatization produces the correct base form, “good.” In many typical implementations, stemming involves lower computational cost because it applies surface-level rules without dictionary access, while lemmatization tends to be more accurate for irregular forms because it draws on lexical knowledge. However, these trade-offs are not universal: the relative speed and accuracy of stemming and lemmatization depend on the specific implementation, the morphological characteristics of the language, and the availability of lexical resources. For morphologically complex languages with limited dictionaries, a well-designed stemmer may outperform a poorly resourced lemmatizer in both speed and effective accuracy.

Stemming is often preferred for tasks involving high-throughput processing of large text volumes, such as search engine indexing, where its typically lower computational cost is advantageous. Hence the choice of whether to use stemming or lemmatization depends on very specific requirements of the application and the language being analyzed. In some cases, a combination of both techniques may be the most effective approach to achieve the desired results. Figure 2 shows different types of stemming algorithms. Stemming methods are broadly divided into two categories: rule-based stemmers and statistical stemmers. Stemming techniques based on predefined linguistic rules remove affixes from words to identify their original root forms and this technique is called Rule Based method. These methods are based on language-specific knowledge. On the other hand, statistical stemming algorithms use probabilistic models and machine learning techniques to identify word roots based on patterns and statistical distributions in a large corpus of text. Rule-based approaches are generally divided into three main categories: Table Lookup, Morphological methods, and Affix Stripping techniques. Statistical algorithms are also divided into three categories: Lexicon-based, Corpus-based, and character n-grams. There are also hybrid stemming methods that use both approaches. Figure 2 illustrates the different types of stemming methods, categorized broadly into rule-based and statistical approaches, with subtypes such astable lookup, morphological analysis, lexicon and corpus analysis, affix stripping, and character n-gram techniques. It is estimated that there are around 7,000 differently languages spoken in the present era, with many of them categorized as resource-poor languages for the scarcity of language technology tools and resources available for their preservation and development (Nathani et al., 2020). We have focused on those languages that are either resource-poor, low-resource, or morphologically rich as defined in Section 2.3, such as languages with a smaller vocabulary or fewer available dictionaries (resource-poor languages). For these types of languages, stemming is more effective at reducing words to their roots, even if the resulting forms are not always accurate. Some of these languages are Arabic, Urdu, Indonesian, Russian, Hungarian, Assamese, Bengali, Bishnupriya Manipuri, Bodo, etc. The majority of the stemming algorithms discussed in this study are rule-based, with only a few examples being statistical-based (supervised and unsupervised approaches). Additionally, a very small number of algorithms are classified as hybrid, combining elements of both rule-based and statistical techniques. There are various use cases for hybrid algorithms, such as KreolStem (Gobin-Rahimbux et al., 2024). It is a stemmer specifically designed for hybrid language-dependent languages (Gobin-Rahimbux et al., 2024).

**Figure 2:**
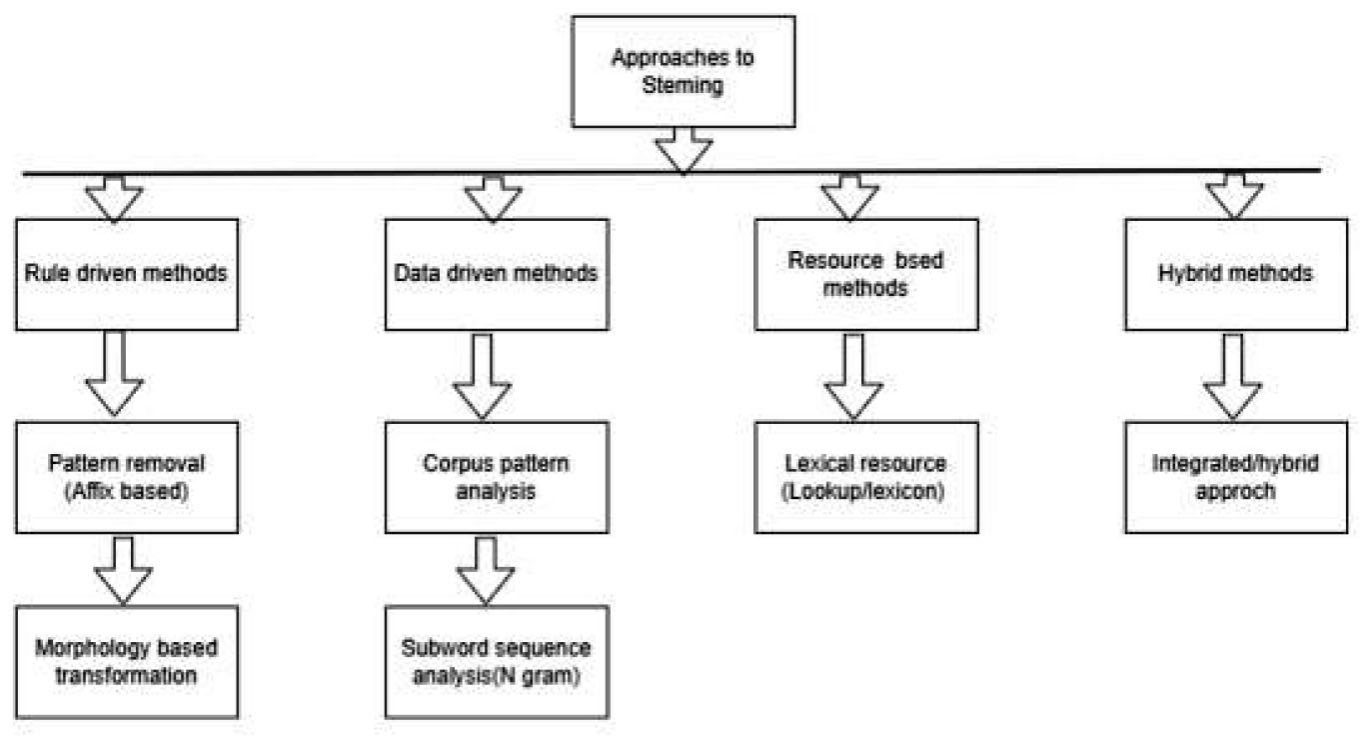
Taxonomy of stemming approaches, categorized into rule-driven, data-driven, resource-based, and hybrid methods.

With the rise of deep learning, stemming is increasingly linked to neural sequence models and subword representations.Transformer-based architectures and character-level neural models have shown strong capability in learning morphological normalization, particularly in low-resource and multilingual settings (Zhu et al., 2019). However, despite these advances, explicit stemming remains critical for languages lacking annotated corpora or pre-trained models, reinforcing the relevance of rule-based and hybrid approaches (Feldman and Sanger, 2007). This motivates a comprehensive reassessment of stemming techniques across diverse linguistic families. Despite the huge work that have been carried on stemming, the existing surveys has some scope to improve and they have mostly focused on a single language family. It has ignored t neural-assisted approaches entirely, or lack a systematic methodology for paper selection. This survey addresses these gaps by (1) systematically reviewing stemming techniques with primary focus on Afro-Asiatic and Indo-Aryan language families—which together account for the largest concentration of resource-poor language stemming research in the literature—alongside selective coverage of Turkic, Uralic, and multilingual settings where directly relevant studies exist; (2) including emerging neural and hybrid paradigms alongside classical rule-based and statistical methods; (3) covering a unified evaluation framework with multi-dimensional metrics; and (4) identifying open challenges and future research directions for low-resource NLP. Based on existing understanding, this survey provides a focused overview of stemming techniques for resource-poor languages and it includes a brief discussion of transformer-based and sub-word-aware approaches.

## 2 Related work

### 2.1 Systematic search methodology

This review follows a structured literature search protocol to ensure reproducibility and minimize selection bias. Searches were conducted across five electronic databases: IEEE Xplore, ACM Digital Library, Scopus, ACL Anthology, and Google Scholar. The database-specific search strings used were as follows:

- IEEE Xplore: (“stemming” OR “morphological normalization”) AND (“low-resource” OR “resource-poor” OR “morphologically rich”)
- ACM Digital Library: (“stemming algorithm” OR “affix stripping”) AND (“low-resource language” OR “morphological analysis”)
- Scopus: TITLE-ABS-KEY (“stemming” AND “resource-poor”) OR (“morphological segmentation” AND “low-resource NLP”)
- ACL Anthology: “stemming” AND (“low-resource” OR “agglutinative” OR “Indo-Aryan” OR “Afro-Asiatic”)
- Google Scholar: “stemming techniques” AND (“resource-poor languages” OR “morphologically rich languages”).

The search covered publications from 1968 to March 2025, with a small number of additional relevant 2025 publications identified through citation tracking of the included studies. Table 1 reports the number of records retrieved per database before deduplication.

**Table 1:** Records retrieved per database during the systematic literature search.

Database	Records retrieved
IEEE Xplore	68
ACM Digital Library	52
Scopus	71
ACL Anthology	63
Google Scholar	56
**Total before deduplication**	**310**
**After deduplication**	**200**

#### 2.1.1 Deduplication

Duplicate records identified across databases were removed using title and DOI matching, reducing the pool from 310 to 200 unique records.

#### 2.1.2 Screening

Two authors independently screened all 200 titles and abstracts against the inclusion and exclusion criteria. Papers were included if they: (a) proposed or evaluated a stemming or morphological normalization technique; (b) targeted at least one resource-poor or morphologically rich language; and (c) were published in a peer-reviewed venue with full text available. Papers were excluded if they focused exclusively on high-resource languages without cross-lingual relevance, or if the full text was unavailable. Peer-reviewed publication was required for inclusion, with one documented exception: a high-relevance arXiv preprint addressing sub-word information transfer for low-resource morphology (Zhu et al., 2019) and a morphologically annotated corpus resource (Akra et al., 2025) were retained where no peer-reviewed equivalent was identified at the time of the search. Disagreements between the two screeners were resolved through discussion and consensus. Where consensus could not be reached, the third author served as arbitrator.

#### 2.1.3 Full-text assessment

Papers passing the title and abstract screen were retrieved in full and assessed independently by two authors against the same inclusion criteria. This stage reduced the pool from 200 to 95 records.

#### 2.1.4 Quality assessment

Each full-text paper was assessed on three dimensions: (1) clarity and reproducibility of the reported methodology; (2) specificity of language coverage, with preference given to papers explicitly targeting resource-poor or morphologically rich languages; and (3) reliability of reported evaluation metrics. Papers failing on all three dimensions, including those for which full text ultimately proved inaccessible upon detailed review, were excluded, reducing the final set to 68 included studies.

#### 2.1.5 Data extraction

A standardized extraction template was applied to all 68 included studies, capturing the following fields for each paper: language family targeted, stemming paradigm employed, dataset used, evaluation metrics reported, downstream application tested, and key findings. This extraction underpins in this review. The PRISMA-style flow diagram illustrating the complete screening process is presented in Figure 3.

**Figure 3:**
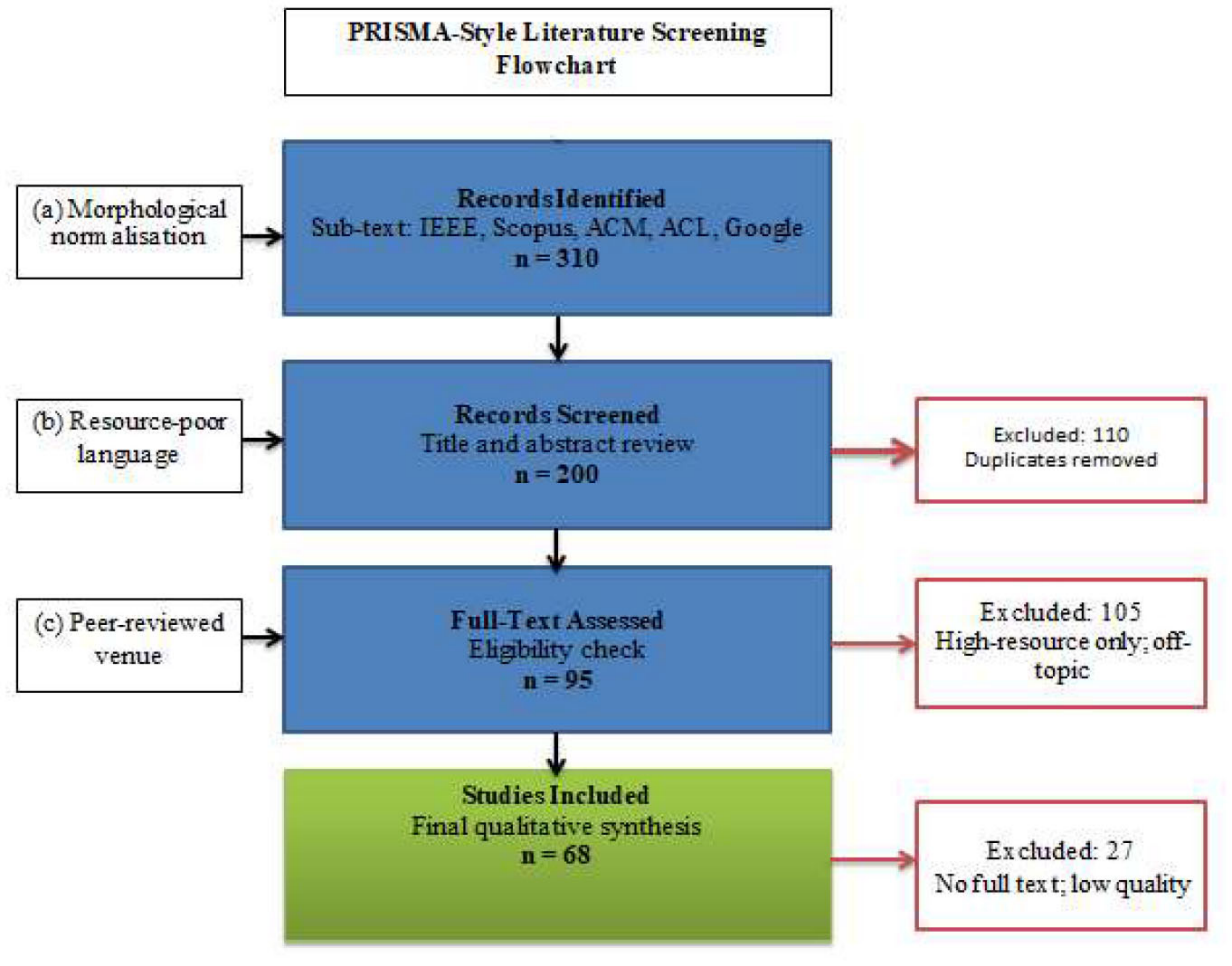
PRISMA style literature screening flowchart.

### 2.2 Scope and conceptual boundaries

This review adopts explicit stemming as its primary unit of analysis. For the purposes of this survey, stemming is defined as any algorithmic process that reduces an inflected or derived word form to a common base form—the stem—through affix removal, morphological rule application, or statistical pattern induction, without necessarily producing a linguistically valid dictionary form. This definition deliberately excludes tasks that are related but methodologically distinct. Table 2 clarifies the conceptual boundaries between stemming and adjacent tasks covered or referenced in this review. Studies in this review are classified as primary if they directly propose, evaluate, or compare a stemming or root extraction method. Studies are classified as adjacent if they employ stemming as a pre-processing component or evaluate stemming indirectly through downstream task performance. Studies involving only sub-word tokenization, G2P conversion, or POS tagging without a direct stemming component are labeled as peripheral and are cited for contextual relevance only, not included in the primary synthesis. Specifically regarding sub-word tokenization: BPE and SentencePiece are discussed in Sections 2.3, 6.5 as implicit morphological proxies that approximate stemming behavior in neural pipelines, but they are not classified as stemmers. Their inclusion is justified by their practical relevance to resource-poor language processing, where explicit stemmers are unavailable. Similarly, the G2P study by Suyanto et al. (2022) is cited in the evaluation section for its use of n-gram taggers and phonotactic rules that interact with Indonesian stemming, not as a stemming study per se.

**Table 2:** Conceptual boundaries between stemming and adjacent tasks covered or referenced in this review.

**Task**	Definition	Relation to stemming
Stemming	Reduces word to stem via affix removal or pattern induction	**Primary focus**
Lemmatization	Reduces word to dictionary base form using morphological analysis and context	Adjacent—compared where relevant
Root extraction	Extracts trilateral or consonantal root (Arabic, Semitic)	Subset—treated as stemming variant
Morphological segmentation	Segments word into morpheme sequence	Adjacent—included where it serves stem identification
Sub-word tokenization (BPE/SPM)	Splits word into frequency-based sub-word units	Adjacent—discussed as implicit stemming proxy; not treated as explicit stemmer
Grapheme-to-phoneme (G2P)	Converts orthographic form to phonemic representation	Peripheral — included only where phonemicization directly informs stem identification
PoS tagging	Assigns grammatical category to tokens	Peripheral—included only where joint PoS–stemming models are evaluated
Word sense disambiguation	Resolves lexical ambiguity using context	Excluded from primary synthesis
General text classification	Assigns category label to document or sentence	Excluded from primary synthesis; stemming impact discussed as downstream effect

### 2.3 Operational definitions

Four terms are used throughout this review that are related but not interchangeable. This section provides explicit operational definitions and clarifies how each term is applied consistently in the classification of languages and studies. Resource-poor language refers to a language for which fewer than three of the following resources exist in publicly accessible form: (1) a tokenised corpus exceeding one million words; (2) a manually annotated morphological or PoS-tagged dataset; (3) a dedicated morphological analyser or stemmer; (4) coverage in a multilingual pre-trained model such as mBERT or XLM-RoBERTa; (5) representation in a shared evaluation benchmark such as Universal Dependencies or SIGMORPHON. Languages satisfying this criterion in this review include Bishnupriya Manipuri, Bodo, Sindhi, and Kreol Morisien. Low-resource language is used as a broader, relative term referring to any language whose available training data and NLP toolchain are substantially smaller than those of high-resource languages such as English, Chinese, or Arabic. A language may be low-resource without being morphologically complex. This term is used in this review when discussing data scarcity in general, without implying a specific morphological profile. Morphologically rich language refers to a language in which words encode a large number of grammatical distinctions through inflection or derivation, resulting in high type-to-token ratios and large vocabulary sizes relative to corpus size. This includes both fusional languages such as Arabic and Turkish and agglutinative languages such as Finnish, Hungarian, and Bengali. Morphological richness increases the importance of stemming because vocabulary sparsity directly degrades the performance of frequency-based NLP models. Agglutinative language is a subset of morphologically rich languages in which morphemes are concatenated sequentially with relatively clear boundaries and minimal fusion. Turkish, Finnish, Hungarian, and Kannada are agglutinative. Arabic and Urdu are fusional and root-based rather than agglutinative, and are classified separately in this review. Table 3 summarizes the classification of all languages discussed in this review according to these four criteria.

**Table 3:** Classification of all languages discussed in this review according to the four operational criteria defined in Section 2.3.

**Language**	Resource-poor	Low-resource	Morphologically rich	Agglutinative
Arabic	No	No	Yes	No (fusional)
Urdu	Partial	Yes	Yes	No (fusional)
Turkish	No	Partial	Yes	Yes
Finnish	No	Partial	Yes	Yes
Hungarian	No	Partial	Yes	Yes
Bengali	Partial	Yes	Yes	Partial
Assamese	Yes	Yes	Yes	Partial
Bishnupriya Manipuri	Yes	Yes	Yes	Yes
Bodo	Yes	Yes	Yes	Yes
Sindhi	Yes	Yes	Yes	No
Indonesian	No	Partial	Partial	No
Kannada	Partial	Yes	Yes	Yes
English	No	No	No	No
Kreol Morisien	Yes	Yes	Partial	No

“Partial” indicates that the criterion is met for some domains or registers but not consistently across available resources.

Table 3 summarizes the classification of all languages discussed in this review according to these four criteria.

“Partial” indicates that the criterion is met for some domains or registers but not consistently across available resources. These definitions are applied consistently in Sections 5, 7, 8 when classifying stemming approaches by their target language profile. When a study covers multiple languages spanning different categories, it is classified according to the most resource-constrained language in its evaluation set. Throughout this review, “resource-poor” is used for languages meeting the operational definition above, while “low-resource” is retained as a general term when referring to data scarcity broadly or when reflecting the terminology of cited studies.

### 2.4 Language sampling strategy and coverage limitations

The language coverage in this review reflects the distribution of stemming research in the existing literature rather than a uniform sampling across all linguistic families. Following the systematic search described in Section 2.1, the majority of identified primary stemming studies target Arabic, Urdu, Bengali, Assamese, Turkish, Indonesian, Finnish, and Hungarian. This concentration is itself a finding of the review: it reveals that stemming research remains heavily skewed toward Afro-Asiatic and South Asian languages, with substantially fewer studies targeting African, Austronesian, Niger-Congo, Dravidian, and Sino-Tibetan language families. Languages were included in this review if at least one primary stemming study meeting the inclusion criteria of Section 2.1 was identified for that language. Languages for which no qualifying study was found were not included in the synthesis, regardless of their resource status. This criterion-based inclusion ensures that coverage claims are grounded in the retrieved literature rather than in assumed representativeness. The following language families receive limited or no systematic treatment in this review due to the absence of qualifying primary studies in the retrieved literature: • Niger-Congo/Bantu languages: Only one adjacent study on morphological clustering of Bantu words was identified (Chavula and Suleman, 2017), which does not meet the primary stemming criterion. • Dravidian languages: One peripheral study on Kannada morpheme segmentation was identified (Bhat, 2012) and one on Gujarati (Dave et al., 2024a). No qualifying Tamil or Telugu stemming study was retrieved. • Austronesian languages beyond Indonesian: No qualifying studies were identified. • African languages beyond Arabic: No qualifying studies were identified. This concentration has two direct consequences that are acknowledged explicitly. First, the conclusions of this review regarding algorithm suitability and evaluation standards are most applicable to Afro-Asiatic and Indo-Aryan languages and should be applied cautiously to other language families. Second, the absence of studies for many language families is itself an open research gap, and future work should explicitly target underrepresented families such as Bantu, Dravidian, and Austronesian languages.

### 2.5 Previous work

Early text processing systems depended on rule-based normalization techniques such as stemming to reduce lexical variability and improve retrieval effectiveness. Foundational algorithms, including the Lovins and Porter stemmers, established suffix-stripping as a practical approximation of morphological analysis. As text classification and information retrieval systems evolved, statistical approaches began to complement linguistic rules by exploiting corpus-level patterns (Said and Ismail, 2025). The emergence of machine learning further shifted focus toward data-driven feature representations, while stemming continued to serve as a lightweight and language-aware pre-processing step. This historical evolution underscores the enduring relevance of stemming, particularly in morphologically rich and resource-poor languages (Siino et al., 2025). The evolution of NLP approaches, categorized by core techniques and representative features has been summarized in the below Table 4. Related work on stemming spans rule-based, statistical, and more recent hybrid approaches, reflecting the evolution of natural language processing from linguistically driven methods to data-driven models. This section reviews foundational rule-based and statistical stemming techniques across multiple languages, followed by emerging trends that integrate sub-word modeling and hybrid learning for resource-poor settings. Stemming is a crucial aspect of Part-of-Speech (PoS) tagging and NLP application. Stemming is the method of shrinking a word to its base or root form to group words that have the same meaning. Rule-based stemmers use linguistic rules to remove affixes from a word and obtain its root form, whereas statistical stemmers employ machine learning algorithms to analyze patterns within a given dataset and subsequently identify the root form of a word based on the frequency of its occurrence. Various rule-based techniques are available in the literature. Moreover, rule-based stemming techniques are used mostly in resource-poor languages, and some of those methods are discussed here. For Indonesian languages, n-grams are used to find similarity between words and reduce error; the dataset is composed of 6,898 documents gathered from the Kompas website and is used for training (Adriani et al., 2007). For stemming Urdu text, six new classes of infix are needed for the Urdu language, along with a minimum word length rule for generating the stem of Urdu text, as used in Ali et al. (2017). For efficiently evaluating any type of stemmer, an automatic method to compare and evaluate Arabic stemmers also looks at various parameters such as accuracy and total time taken in execution (Jaafar et al., 2017). In the Turkish language, the stemming algorithm FINDSTEM is used for Turkish documents and shows good accuracy. Inflectional and derivational stemmers are used in the FINDSTEM and AF algorithms. Inflectional rules are only handled by L-M (Sever and Bitirim, 2003). For Arabic, three types of APIs are available that can be used for stemming, syntactic parsers, and morphological analyzers, which are available on the SAFAR platform (Jaafar and Bouzoubaa, 2015). There are two stemming methods for the Arabic language that are based on the light stemming method. Two predefined lists can be used, not only one as most of the methods do. Also, using a POS tagger somewhat reduces the burden of stemming (Mustafa et al., 2019). Some of the statistical techniques are described here, such as phonemicization, also known as grapheme-to-phoneme conversion (G2P), which is a method of translating a word into its pronunciation (Suyanto et al., 2022). The key principle is that in Information Retrieval systems, words with comparable meanings and morphologies should be treated as equivalent. As a result, the major goal is to minimize all morphological divisions to the word's root form (Alotaibi and Gupta, 2018). For the Kannada language, detecting a morpheme is a problematic task due to the highly inflectional and agglutinative morphology of this language. An unsupervised algorithm can be used for morphological segmentation. Training of the algorithm is usually accomplished using a corpus of 990 thousand words. The algorithm shows an F-measure of 73% Bhat, (2012). For brief text clustering, Yin and Wang (2014) propose that a collapsed Gibbs sampling approach for the Dirichlet Multinomial Mixture model (GSDMM) can be used. GSDMM is fast, and it can handle high-dimensional issues of short texts and discover representative words for each cluster. Removing stop words and using proper weighting techniques play an important role. Techniques such as Latent Semantic Indexing (LSI) and Latent Dirichlet Allocation have already been used to study corpora. There are different weighting methods that can improve the results (Wilson and Chew, 2010). When dealing with extremely brief texts such as social media messages, traditional topic models face a serious sparsity problem. While Dirichlet Multinomial Mixture (DMM) can deal with sparsity, it is still particularly sensitive to ordinary and frequent words (Li et al., 2019). Stemming can be done using co-occurrence statistics; this type of method uses statistics derived from the co-occurrence between two word variations. Co-occurrence determines how frequently a pair of word types appears in a text as well as throughout the whole corpus (Paik et al., 2011). Some stemming methods use the HMM model for rich inflectional languages such as Assamese. In that case, a combination of a rule-based algorithm for the prediction of single-letter suffixes and an HMM model for the prediction of single-letter suffixes is used. This combination achieves strong stemming accuracy for Assamese (Saharia et al., 2013). To identify the stem of Bangla words, the n-gram language model can be used; for this, the 6-gram model achieves around 40% accuracy (Urmi et al., 2016). Recent literature indicates a shift from purely rule-based and statistical stemmers toward neural and hybrid morphological processing frameworks. Multilingual pre-trained models leveraging shared sub-word vocabularies have demonstrated improved generalization for unseen or low-resource languages (Zhu et al., 2019). Nevertheless, such approaches often require substantial computational resources and annotated data, limiting their applicability in truly resource-poor contexts (Zhu et al., 2019). Consequently, hybrid stemming models that combine linguistic rules with lightweight statistical learning have emerged as a promising research direction (Bölücü and Can, 2019). Recent state-of-the-art approaches increasingly rely on transformer-based architectures that implicitly capture morphological regularities through sub-word tokenization and contextual representation learning. Such models reduce explicit dependence on handcrafted stemming rules by learning morphology-aware embeddings, particularly in multilingual and cross-lingual settings. However, their effectiveness in truly resource-poor languages remains constrained by data availability and computational requirements, thereby preserving the relevance of classical and hybrid stemming approaches.

**Table 4:** Evolution of NLP approaches.

Category	Core approach	Typical features	Representative era
Rule-based	Manually crafted linguistic rules	Keywords, patterns, lexicons	Early NLP
Statistical/ML	Probabilistic and classical ML models	BoW, TF-IDF, n-grams	1990s–2010s
Deep learning	Neural architectures	Word embeddings, CNN/RNN features	2015–2020
Transformer-based	Attention-driven pre-trained models	Contextual embeddings, sub-words	2018–present
Hybrid	Rule-based + data-driven models	Linguistic rules + learned features	Current/Low-resource NLP

## 3 Applications of stemming in NLP

Information Retrieval (IR) is a key area where stemming plays an important role, particularly in search engine systems. Earlier, before the rise of blogs and social media platforms, news organizations widely shared their headlines across various websites using IR-based distribution methods. These data may contain a wealth of useful information relevant to a variety of social research fields (Dilrukshi et al., 2013). According to Chen and Gey (2002), one strategy for cross-language retrieval was to translate English queries into Arabic using online English–Arabic machine translation systems and then use root retrieval to locate the verb root. Without utilizing any language rules, this technique was able to extract suffixes and prefixes (Azman, 2019). There is a necessity to improve Arabic Information Retrieval techniques using stemmers and lemmatizers; therefore, a stemmer has been proposed to address this issue by Zeroual and Lakhouaja (2017). Paik et al. (2011) proposed a novel language-independent stemming algorithm that is well suited for information retrieval. This algorithm has three key characteristics: it is effective in retrieving information, versatile in its applications, and computationally efficient. The algorithm has been tested on several languages and shows variation with morphological complexity. In experiments using the TREC-2001 dataset, different lightweight heuristic-based stemmers were developed along with a statistical co-occurrence–based stemmer for Arabic information retrieval, and their retrieval performance was evaluated in comparison with a morphological analyzer (Larkey et al., 2002). The recommended approach is to compare the results of multiple Arabic root extraction methods. Deriving root forms of words plays an important role in many natural language processing tasks, including information retrieval and machine-based language understanding. Stemming helps during text classification in terms of model accuracy. Many research works employed stemming. Some of them are discussed here. According to Krishnakumar (2006), as online information has surged vert fast, there is an increasing requirements for tools that aid in the discovery, filtering, and management of high-volume data. Construction of text classifier manually is extremely costly, making unsupervised text classification increasingly popular. Unsupervised morphological segmentation has been found to be an attractive solution for languages with little linguistically available data, such as most of the Bantu languages. The paper suggests a model that uses a weighted similarity measure to determine the weights of the Ordered Weighted Aggregator (OWA) operator, using the normal distribution (Chavula and Suleman, 2017). A difficulty with the categorization of Arabic documents is highlighted. At the moment, the Vector Space Model (VSM) is a common way of describing text features in text categorization. TF-IDF weights were adopted to calculate the weight of each term along every dimension of the model of the text feature (Wang et al., 2010). The implications of the light stemming approach on feature extraction for Arabic document categorization using Bag of Words (BoW) and Term Frequency-Inverse Documents (TF-IDF). The classification model is built with two methods i.e., using Support Vector Machine (SVM) and K-nearest Neighbor (kNN) models (Alyousf and Alhalabi, 2025). A method for removing suffixes is suggested, and GRAS(GRAph-based Stemmer) is appropriate for languages that use suffixes since it groups similar word forms based on suffix information from the given text (Paik et al., 2011). The “Quranic Arabic Corpus” is a richly annotated linguistic dataset that encodes multiple layers of information, such as grammatical tagging and sentence structure analysis based on dependency relations (Akra et al., 2025). Alnaied et al. (2020) have proposed a different way to extract the stem from Arabic text by applying a set of rules using the AMIR dictionary. Abstract Arabic Information Retrieval (AMIR) is a solution to problems related to web and social media networks in Arabic text. Maximum Likelihood training is a procedure that estimates Hidden Markov Model (HMM) parameters from training data, but it does not guarantee an improvement in tagging accuracy (Merialdo, 1994). Stemming is performed by estimating the most likely route using HMM states. This approach discovers the HMM parameters, which are then used to estimate the most likely stem for every input (Melucci and Orio, 2003). A common approach is proposed to define a model and enhance the likelihood of the latent structure inferred from the observed data. This is achieved using maximum-likelihood estimation (MLE) of the model parameters by Goldwater and Griffiths (2007). With the help of dictionary lookup and customized rules for the Gujarati language, more attention is directed toward prefix removal in this language by Dave et al. (2024a). In Indonesian POS tagging problems, Yuwana et al. (2018) evaluate deep architectures to identify the most effective structures. Various models with different numbers of hidden layers are investigated. Using stemming techniques in sentiment analysis, the number of computations required becomes less, as stemming reduces all words to their root forms. As stated by Balahur and Turchi (2014), sentiment analysis is a task in natural language processing that involves detecting and classifying sentiments in texts. When preparing a text for mining, stemming is employed to revert words to their original roots, enabling more efficient processing of natural language in subsequent stages. Link detection is a constantly growing approach to text analysis that shares and expands on many of the main aspects of text mining, as well as new tools for individuals to better understand textual data resources (Krishnakumar, 2006). An unsupervised program that learns the structure of words by itself was proposed, in which raw untagged Hindi corpus data are fed to the program to help it discover all possible morphological variants of a word (Krishn et al., 2012). In Table 5, the proposed survey provides broader coverage than recent surveys on stemming for resource-poor languages. The older survey papers are more focused on the Urdu language, in which Jabbar et al. (2019) described approaches and challenges in Urdu stemmers. A well-defined architecture is also shown and described. On the other hand, this survey paper is concerned with several languages in stemming. To obtain reliable findings, many evaluation measures are applied. There are also benefits and downsides. Multilingual text processing is advantageous because different languages have varied information content. Text mining software and information extraction tools have been developed for various languages, but their availability has been limited to a few languages due to the significant development costs involved (Jabbar et al., 2019). Because of the massive quantity of information individuals have access to, which continues to grow as new technologies and the Internet are used, improved information retrieval methods have become vital. Stemming is one of the most important approaches for improving the accuracy and performance of an information retrieval system (Steinberger, 2012). There are several applications of NLP such as information retrieval, part-of-speech tagging, parsing, and machine translation that require the use of stemming process. It is a process in morphology that simplifies words by stripping their inflectional variations and reducing them to a common root form (Anam et al., 2024). Stemming is a text-mining preprocessing procedure that is often used in Natural Language Processing (NLP). A stemmer can change morphologically similar words to root words without having to undertake morphological analysis of that term (Purohit and Yogi, 2025). Beyond traditional information retrieval and text classification, stemming has proven beneficial in sentiment analysis, hate speech detection, topic modeling, and document clustering. By reducing lexical sparsity, stemming improves feature consistency and enhances classifier robustness, particularly in morphologically complex languages (Alshalabi et al., 2022b). Recent studies further show that stemming contributes to improved semantic similarity estimation and topic coherence when combined with probabilistic topic models (Medvecki et al., 2023). These findings reinforce the role of stemming as a foundational preprocessing step in modern NLP pipelines.

**Table 5:** Comparison of the proposed survey with recent surveys on stemming for resource-poor languages.

Feature	This survey paper	Recent surveys (Dave et al., 2024b; Alyousf and Alhalabi, 2025; Pakray et al., 2025)
Languages covered	Several languages are covered such as Assamese, Urdu, Arabic, Turkish, Finnish, English, etc.	Indian and non-Indian vernacular languages; general low-resource languages
Application domains	IR, PoS tagging, text classification, morphological analysis, lemmatization	Primarily IR and general NLP applications
Stemming method coverage	Rule-based, statistical, unsupervised, hybrid, and neural-assisted methods	Mainly rule-based and algorithmic methods; limited neural discussion
Architecture/taxonomy	Detailed taxonomy and workflow diagrams included	No explicit architectural or workflow diagrams
Evaluation measures	Accuracy, Precision, Recall, F-measure, IR metrics, linguistic error analysis	Mostly accuracy-based and IR-centric metrics
Resource-poor language focus	Explicit, systematic, and central to the study	Moderate to high-level discussion, often task-dependent
Advantages/limitations	Both strengths and limitations critically analyzed with future research directions	Limited critical analysis of limitations
Language sampling transparency	Explicit criterion-based inclusion with acknowledged concentration on Afro-Asiatic and Indo-Aryan	Language selection criteria not stated

### 3.1 Synthesis across application domains

Across the literature, information retrieval consistently proves the largest and most measurable benefit from stemming, particularly for Arabic and Urdu, where vocabulary reduction through affix stripping directly improves MAP scores. Text classification shows moderate gains, with the benefit highly dependent on the morphological complexity of the target language—agglutinative languages such as Turkish and Finnish show greater improvement than analytic languages. Sentiment analysis benefits are mixed: stemming reduces sparsity but occasionally collapses semantically distinct forms, introducing noise in fine-grained opinion tasks. Topic modeling and document clustering show consistent improvement when stemming is combined with probabilistic models such as LDA and DMM, primarily through better term frequency estimation. Notably, stemming's role in low-resource machine translation remains underexplored and represents a significant gap in the current literature.

## 4 Generic architecture

The generic stemming pipeline, illustrated in Figure 1, proceeds through six stages. Raw text input is drawn from diverse sources—web and social media, news corpora, annotated treebanks, domain documents, and religious texts—and passed to a preprocessing stage comprising language detection, tokenization, normalization, stop-word removal, morpheme segmentation, and noise removal. The preprocessed tokens then enter one of four parallel stemming paradigms: rule-based affix and lookup stemming, guided by a handcrafted lexicon or morphological grammar; statistical n-gram stemming, driven by corpus-derived frequency distributions; hybrid PoS-HMM stemming, combining rule-based and statistical components sequentially or in parallel; and neural transformer stemming, which learns morphological regularities directly from data. After stem extraction, a post-processing validation step checks the candidate stem against a dictionary or minimum length constraint to avoid over-stemming. Output from these paradigms feeds into a modeling stage that draws on classical NLP representations (TF-IDF, n-grams, HMM/CRF, and rule-based features), deep learning representations (Word2Vec, FastText, CNN/BiLSTM, and morphology-aware embeddings), or transformer-based representations (BERT, mBERT, XLM-R, and large language models). The resulting stems and representations are assessed using intrinsic metrics (precision, recall, F-measure, and UI/OI/VRR), extrinsic information-retrieval metrics (MAP, precision@k, and NMI), and standard benchmarks (TREC, FIRE, Universal Dependencies, SIGMORPHON, and SemEval). The pipeline concludes with downstream applications, including search, text classification, sentiment analysis, question answering, machine translation, and iterative refinement of the stemming rules themselves. Modern pipelines optionally incorporate a sub-word encoding layer—such as Byte Pair Encoding (BPE) or SentencePiece—between tokenization and stemming, allowing the system to handle out-of-vocabulary words in low-resource settings (Zhu et al., 2019). Modern stemming pipelines increasingly incorporate statistical or neural components alongside traditional affix-stripping modules. Morfessor 2.0, an unsupervised morphological segmentation tool, can be integrated prior to root extraction.

Unsupervised morphological2.0 can be integrated prior to root extraction to identify latent morpheme boundaries (Özbey and Karcili, 2021). Additionally, character-level or transformer-based encoders may be employed to refine stem predictions in ambiguous cases, particularly for highly inflected languages (Zhu et al., 2019). This modular architecture enables flexible adaptation across languages with varying resource availability.

## 5 Stemming algorithms for resource-poor languages

Recent advances highlight the growing role of unsupervised and weakly supervised learning in stemming for resource-poor languages. Sub-word-based statistical models and joint learning frameworks that simultaneously perform stemming and part-of-speech tagging have demonstrated superior performance compared to standalone stemmers (Bölücü and Can, 2019). However, their effectiveness remains constrained by data scarcity and morphological irregularities. As a result, rule-based and hybrid stemmers continue to dominate for resource-poor and morphologically rich linguistic settings as defined in Section 2.3. Table 6 summarizes recent benchmark datasets and shared-task resources relevant to stemming for resource-poor languages.

**Table 6:** Recent benchmark datasets and shared-task resources relevant to stemming for resource-poor languages.

Dataset/benchmark (Year)	Coverage	Annotation/task	Stemming relevance
Universal dependencies (UD) v2.14 (2024)	161 languages	PoS, dependency, morphology	Indirect stemmer evaluation
SIGMORPHON 2024: morphological inflection	Multi-language	Low-data inflection	Low-resource benchmarking
SIGMORPHON 2024: sub-word tokenization	Multi-language	Subword tokenization	Stemmer–sub-word comparison
SIGMORPHON shared-task repositories (2024)	Multi-language	Splits, Codes and baselines	Reproducible evaluation
Canonical morpheme segmentation (2025)	Low-resource	Morpheme segmentation	Stem-adjacent proxy
DUDU ottoman Turkish treebank (2025)	Ottoman Turkish	UD-style annotation	Historical portability

Sources: Universal Dependencies (Nivre et al., 2020); SIGMORPHON 2024 Subword Tokenization (Li, 2024); Canonical Morpheme Segmentation (Beekhuizen, 2025); and DUDU Ottoman Turkish Treebank (Yılandiloğlu and Siewert, 2025).

As mentioned above, in this study, different types of stemming algorithms are analyzed. These algorithms can be classified as rule-based, supervised, and unsupervised. Many of them work well, but there is still some work required in areas such as dealing with highly inflected languages. In Table 7, various stemming algorithms are shown with respect to their type and language coverage.

**Table 7:** Comparison of representative stemming methods discussed in the literature.

References	Method	Type	Description	Languages	Classification
Ali et al. (2017)	Urdu stemmer and short text classification	Rule-based	Short-text stemming	Urdu, Arabic	Primary
Alotaibi and Gupta (2018)	Language-independent stemmer for IR	Unsupervised	IR normalization	Multilingual	Primary
Jaafar et al. (2017)	Improved Arabic stemming method	Rule-based	Lexicon validation	Arabic	Primary
Alnaied et al. (2020)	Arabic Morphology Information Retrieval (AMIR)	Rule-based	Dictionary extraction	Arabic	Primary
Nathani et al. (2020)	Linguistica 5 (MDL-based)	Unsupervised	MDL stem discovery	Sindhi	Primary
Bölücü and Can (2019)	Bayesian HMM for joint PoS tagging and stemming	Unsupervised framework	Joint PoS–stemming	Turkish, Finnish, Hungarian, Basque, English	Primary
Özbey and Karcili (2021)	Morfessor 2.0	Unsupervised statistical model	Morpheme segmentation	Turkish	Primary
Alshalabi et al. (2022b)	Arabic light-based stemmer (DLight)	Rule-based	Affix removal	Arabic	Primary
Singh and Gupta (2019)	Lexical and co-occurrence based stemming	Unsupervised	Lexical normalization	English, Marathi, Bengali, Hungarian	Primary
Saharia et al. (2013)	HMM-based hybrid stemmer	Hybrid approach	Hybrid normalization	Assamese, Bengali, Bodo, Bishnupriya Manipuri	Primary
Alshalabi et al. (2022a)	Broken Plural Rule (BPR) algorithm	Rule-based	Root identification	Arabic	Primary
Jabbar et al. (2019)	Urdu Text Stemmer (UTS)	Rule-based	Multi-stage stemming	Urdu	Primary
Suyanto et al. (2022)	G2P + n-gram tagger	Statistical	Statistical	Peripheral	

Recent advances highlight the growing role of unsupervised and weakly supervised learning in stemming for resource-poor languages. Sub-word-based statistical models and joint learning frameworks that simultaneously perform stemming and part-of-speech tagging have demonstrated superior performance compared to standalone stemmers (Bölücü and Can, 2019). However, their effectiveness remains constrained by data scarcity and morphological irregularities. As a result, rule-based and hybrid stemmers continue to dominate for low-resource linguistic settings.

### 5.1 Unspervised and hybrid learning–based stemming algorithms

#### 5.1.1 Afro-asiatic (semitic) language family

This section reviews supervised, weakly supervised, and hybrid stemming approaches, where supervision is provided through annotated corpora, lexical resources, or benchmark-driven training and evaluation. Alnaied et al. (2020) propose AMIR (Arabic Morphology Information Retrieval), a method to produce a powerful tool to extract Arabic root words by deploying morphological analysis with the help of linguistic rules. AMIR (Arabic Morphology Information Retrieval) performed better in Arabic stem extraction compared to previous methods such as LUCENE (Apache Lucene) and FARASA. The current methods excel at removing affixes but struggle to determine whether the removed letters are essential parts of the root word. The FARASA algorithm effectively segments Arabic text into words by handling prefixes and suffixes. In contrast, the AMIR solution also addresses infixes, thereby expanding its capabilities. The TREC community's standard tool for assessing IR (Information Retrieval) systems is the *trec*_*e*_*val* program (Text REtrieval Conference evaluation tool) (Alnaied et al., 2020). The use of statistical measures as assessment metrics, such as the *TREC*_*E*_*VAL* tool, is employed to quantify accuracy@10, precision@20, and MAP (Mean Average Precision). In terms of frequency metrics, TF-IDF (Term Frequency–Inverse Document Frequency) is also crucial, as it counts the frequency of words used and evaluates the retrieval performance quality of the proposed work. Using the BM25 (Best Matching 25/Okapi BM25) model, AMIR achieved MAP values of 0.34 for 50 queries, whereas LUCENE, FARASA, and no stemmer achieved 0.27, 0.28, and 0.21, respectively. To assess the quality of scheme performance, AMIR was compared against two existing stemmers, FARASA and LUCENE, as well as a no-stemmer baseline using the BM25 model and the LM (Language Model) using the Dirichlet approach. For instance, consider an example from the paper: an Arabic word processed by both AMIR and FARASA/LUCENE, where the word Mosques indicates a plural form. The AMIR method is able to reduce this to the singular form (AMIR rule No. 3), whereas FARASA and LUCENE are unable to reduce it to the singular form, as these stemmers cannot handle plural forms using infixes. Alshalabi et al. (2022b) proposed an improved light-based method that uses a proposed list of suffixes and prefixes. In the algorithm, many rules are applied to a word depending on its size and are able to extract even multiple roots from both long and short Arabic words. Stemmers such as Condlight, ARLST, and Light10 have been utilized for performing the comparative analysis. The proposed stemmer achieves an F-measure of 68 percent, whereas the comparative models achieved a lower F-measure. The mathematical equation for the F-measure is given below.


$$\begin{array}{l} F\text{-measure}=\frac{2\times \left(\text{Precision}\times \text{Recall}\right)}{\text{Precision}+\text{Recall}} \end{array}$$ (1)

After performing the experiments, the proposed DLight stemmer achieved F-measure gains reported as “34% more, 31% more, and 25% more” than the Light, Condlight, and ARLST stemmers respectively, on the TREC 2002 Arabic newswire collection Alshalabi et al. (2022b); the original study does not specify whether these are relative or absolute percentage differences. Several studies have been carried out, but very few focus on words that contain two letters (Alshalabi et al., 2022b). For instance, the extracted root for the word love in the pattern according to various stemming methods will be, which is an incorrect root. To tackle these issues and discover the proper root words, subrules are constructed and a subrule algorithm is created. Here, the Text Retrieval Conference (TREC 2002) is used as the dataset. The BPR (Broken Plural Rule) algorithm contains four steps (Alshalabi et al., 2022a). First, it normalizes the characters; second, it removes Arabized and stop words; third, it removes the article; and finally, it performs a different set of procedures based on the word length. Although supervised stemmers achieve high accuracy in languages with sufficient annotated resources, their performance degrades significantly in low-resource environments. Recent work explores lightweight neural stemmers trained on limited data using character-level supervision, yielding promising results for morphologically rich languages. Nonetheless, the dependence on labeled corpora restricts their widespread adoption, underscoring the importance of rule-based alternatives for Indo-Aryan and Afro-Asiatic languages (Ashraf et al., 2023).

#### 5.1.2 Turkic language family

Özbey and Karcili (2021) proposed the Dual Dictionary Model (SDDM/PDDM) as an extension of Morfessor 2.0, an MDL-based unsupervised stemmer designed for Turkish. Unlike the original Morfessor, which maintains a single codebook, SDDM maintains separate prefix and suffix dictionaries, reducing false-positive stems. The PDDM variant further assigns probabilities to prefix-suffix pairs, identifying the most likely stem boundary. Evaluated on mixed, highly inflected verbal, and uninflected Turkish word sets, PDDM with threshold = 2 and 10 EM iterations outperformed both SDDM and the original Morfessor. Time complexity for stem boundary detection is O(M) in SDDM compared to O(M^2^) in Morfessor, making it significantly more efficient for large Turkish corpora (Özbey and Karcili, 2021).

#### 5.1.3 Multi-lingual

Alotaibi and Gupta (2018) proposed a cognitively inspired, unsupervised, language-independent stemming algorithm that operates without any language-specific knowledge or human supervision. The method estimates both structural and lexical distance between word pairs, removes stop words, and iteratively clusters morphologically related words using a nearest-neighbor merging strategy. Evaluated across English, Bengali, Marathi, and Hungarian using CLEF, TREC, and FIRE test collections with the IFB2 weighting model and TERRIER retrieval system, the method outperformed both corpus-based and rule-based stemmers in MAP across nearly all language-task combinations. Bölücü and Can (2019) extended multilingual stemming by jointly learning Part-of-Speech tags and stems in a fully unsupervised Bayesian HMM framework, demonstrating that joint modeling outperforms standalone PoS taggers and stemmers for agglutinative languages including Turkish, Finnish, and Hungarian, with improvements measured using many-to-one accuracy, NMI, and variation of information metrics (Bölücü and Can, 2019).

#### 5.1.4 Indo-Aryan language family and Uralic language family

Nathani et al. (2020) used a program called “Linguistica 5” that is based on the Minimum Description Length (MDL) method to create an unsupervised learning of morphology. This method is different from the previous rule-based, time-consuming methods. Maximum affix length, Minimum Stem length, and Minimum Signature count will be the emphasis of this stemmer. Through a List of Signatures, the “Linguistica 5” tool will try to find a new signature that will contain a stem and pair of suffixes with every iteration till no new modification is found in the signature. The MDL algorithm that is used in this scenario is the basis of statistical modeling, pattern identification, and machine learning. Minimum Description Length holds a better explanation of a given set of observed data. It helps in the smallest description of the given data. The “Linguistica” command line interface has several options, including Maximum affix length = 4, Minimal Stem length = 4, and Minimum Signature count, which is the minimum number of stems for a valid signature is 5. In this, evaluation process, the proposed algorithm performs well with great accuracy but with some minor ups and downs in the form of under-stemming and over-stemming (Nathani et al., 2020).

#### 5.1.5 Resource-poor languages

Saharia et al. (2013) focused on eastern Indian languages that don't have many resources such as Bengali, Bishnupriya Manipuri, and Bodo. Here an HMM-based hybrid is proposed, to remove the suffixes, rule-based method is employed. Sometimes stemmer does over and under-stemming, to solve this issue dictionary has been used which contains the most frequent words. The proposed stemmer achieves 94% accuracy, 87% accuracy, and 82% accuracy for Assamese, Bengali, Bishnupriya Manipuri, and Bodo respectively. This algorithm is capable of efficiently extracting stem words from inflected words of any length.

## 6 Evaluation of stemming algorithms

### 6.1 Evaluation metrics

There are numerous methods for determining how well a stemmer performs. Methods differ depending on the type of stemmer. Some of the evaluation methods are discussed further below. Among the approaches that are frequently used are frequency count, n-gram (Cavnar et al., 1994), Hidden Markov Models (Rabiner and Juang, 2003), and link analysis. The n-gram tagger is evaluated in the phonotactic domain without the use of a stemmer or phonotactic rules. PERs produced by all G2P models using those optimal settings, as well as comparisons with Transformer-based G2P models, are also considered. Many methods, such as the TERRIER retrieval system, can be used to evaluate a single stemmer. These include: (1) weighting model IFB2, (2) statistical divergence, (3) average retrieval precision, (4) recall accuracy, (5) precision@10, (6) precision@20, and (7) the *t*-test. Comparing the proposed stemmers with previous works helps demonstrate their contributions and improvements. The Buckwalter morphological analyzer and tri-literal root extraction are two evaluation methods used in various experiments. Metrics such as Precision@10, Precision@20, and Mean Average Precision (MAP) are commonly used in the field of information retrieval. The Pearson correlation coefficient between machine scores and human judgments is sometimes used to assess performance. Two evaluation metrics, normalized mutual information (NMI) and purity, are used in document clustering evaluation. Cross-validation (Buckland and Gey, 1994) is a common method for assessing precision and classification performance. Inflectional postfixes are utilized to study the processing of inflectional endings in dialects such as French verbs, yielding notable impacts on various morphological operations and the understanding of morphemes.

Empirical studies reveal that relying on a single evaluation metric can lead to misleading conclusions about stemmer performance. Comprehensive evaluation frameworks now combine intrinsic metrics such as precision, recall, and F-measure with extrinsic measures based on downstream task performance (Lakshmi and Kumar, 2014). Additional indicators, including under-stemming and over-stemming rates, vocabulary reduction ratios, and semantic similarity preservation, provide deeper insight into stemmer behavior (Alhawarat et al., 2021). Such multi-dimensional evaluation is essential for fair comparison across languages and algorithms. Recent benchmark resources increasingly emphasize morphology-aware evaluation through standardized treebanks and shared tasks. In particular, UD v2.14 provides broad multilingual morphosyntactic annotations suitable for cross-language analysis, while SIGMORPHON 2024 tasks offer controlled low-data settings for benchmarking morphology-related components such as stemming, lemmatization, and sub-word tokenization.

### 6.2 Reporting gap in existing literature

Although under-stemming index (UI) and over-stemming index (OI) are formally defined and theoretically important, the majority of studies reviewed in this survey report only accuracy, F-measure, or MAP, without stemming-specific error indices such as UI or OI. This omission limits the ability to diagnose the qualitative nature of stemming errors. Future work should adopt a standardized reporting format that includes UI, OI, VRR, and at least one downstream task metric simultaneously.

### 6.3 Existing stemmers for resource-poor languages

To ensure that the proposed method is superior and provides higher accuracy, it must be compared with its competitors. Several approaches and alternative comparison methods are discussed further below. The proposed minimum word length rule was examined first, followed by the prefix rule, and finally, the infix rule was evaluated. The proposed approach was compared with a lightweight Urdu stemmer. Experiment 1 used the same news headline, and it was discovered that incorrect interpretation of prefixes and postfixes has a significant impact on stemming accuracy (Ali et al., 2017). To classify approaches for evaluating the proposed short Urdu text classification, the following news headlines were used: politics, sports, terrorism, and weather. Their novel infix stemming method and affix stemming techniques significantly enhance categorization accuracy compared with Urdu text classification systems based on Naive Bayes and SVM. The proposed full Urdu text preprocessing and categorization system significantly outperforms competing approaches (Ali et al., 2017).

In terms of PER and WER for Indonesian phonemicization, the model is compared with the state-of-the-art Transformer-based G2P model. There are additional requirements (Alotaibi and Gupta, 2018) when working with language-independent tools that support multiple languages, such as the TERRIER retrieval system, IFB2 weighting model, probabilistic divergence, average retrieval precision, recall precision, precision@10, and the *t*-test. Stanford CoreNLP is also a suitable choice for multilingual tasks. Several light stemmers are available for Arabic, including Light1, Light2, Light3, Light4, Light5, Light6, Light7, Light8, and Light10. In terms of retrieval performance, the Light10 stemmer outperforms the other light stemmers (Larkey et al., 2002). For Arabic information retrieval, FARASA and LUCENE are used, and Jaccard similarity is employed to measure similarities between sets. It is defined as the size of the intersection divided by the size of the union of two sets. When dealing with agglutinative languages, there are primarily three methods for evaluation. (1) Word-based Bayesian HMM: a Bayesian Hidden Markov Model used to determine the probability distribution across a sequence, where states are hidden and based on words. (2) Brown clustering: a technique for grouping related words that creates a hierarchical word cluster. (3) In the anchor HMM model, each hidden state has at least one observation that can only be generated by that state. For example, “they” can only be tagged as a determiner and will not be tagged otherwise. SDDM (Strict Dual Dictionary Model) is a stemming algorithm that approves suffixes to reduce the number of false-positive stems generated by the Naive Dictionary Model (NDM) (Özbey and Karcili, 2021). Furthermore, the Probabilistic Dual Dictionary Model (PDDM) defines a mechanism for determining the most significant stem boundary by calculating the probabilities of all valid prefix–suffix pairs. The Sastrawi technique is improved by employing dynamic affixes to analyze non-formal Indonesian containing abbreviated and distorted words, as well as by developing and testing a text classification model using the Support Vector Machine approach.

### 6.4 Dataset

Table 8 summarizes the benchmark datasets used across the reviewed studies. For each dataset, the language, primary task, and public availability are noted.

**Table 8:** Overview of multilingual NLP datasets.

Dataset	Language	Primary task	Publicly available
TREC 2002 AFP Newswire	Arabic	Information retrieval	Yes, registration required
Urdu News Headline Corpus	Urdu	Text classification	Yes (Open Access)
FIRE 2010	Bengali, Marathi (subset)	IR	Yes, registration required
Wall Street Journal	English	IR, classification	Licensed
KBBI Dictionary	Indonesian	Stemming validation	Yes
Turkish Wikipedia Lexicon	Turkish	Unsupervised stemming	Yes
Quranic Arabic Corpus	Arabic	PoS, Morphology	Yes
EveTAR	Arabic (MSA, dialectal)	Microblog IR	Yes, registration required
SemEval-2017 (Task 4)	English, Arabic	Sentiment analysis	Yes
Universal Dependencies v2.14	150+ languages	Morphology, PoS	Yes
SIGMORPHON 2024	Multi-language	Morphological inflection	Yes

Version and source details for each dataset are as follows: TREC 2002 (NIST/TREC, AFP Arabic Newswire subset); Urdu News Headline Corpus (compiled corpus, see Ali et al., 2017); FIRE 2010 (Forum for Information Retrieval Evaluation, 2010 edition; Bengali and Marathi subsets as evaluated in the cited studies); Wall Street Journal (LDC Catalog No. LDC2000T43); KBBI (Kamus Besar Bahasa Indonesia, official online edition); Turkish Wikipedia Lexicon (2021 Wikipedia dump, tokenised); Quranic Arabic Corpus (v0.4, quran.corpus.org); EveTAR (2016 release, Arabic microblog test collection); SemEval-2017 Task 4 (Twitter sentiment analysis, English and Arabic); Universal Dependencies (v2.14, 2024 release); and SIGMORPHON (2024 shared-task release).

### 6.5 Neural-assisted stemming: an emerging direction

#### 6.5.1 Neural and LLM-assisted approaches to stemming

Neural methods relevant to stemming span several distinct task formulations that are frequently conflated in the literature. This section distinguishes them explicitly, since each produces different outputs and carries different requirements for resource-poor languages.

#### 6.5.2 Character-level sequence-to-sequence stemmers

These models treat stemming as a character-level transduction task, mapping an inflected surface form to its stem. Encoder-decoder architectures with attention have been applied to morphological transduction in the SIGMORPHON shared tasks, showing competitive performance under low-data conditions when augmented with data hallucination or copy mechanisms. Their principal constraint is the requirement for character-aligned training pairs, which do not exist for most truly resource-poor languages.

#### 6.5.3 Neural lemmatization and canonicalization

Distinct from stemming, neural lemmatizers such as those built on the UDPipe and Stanza frameworks generate dictionary base forms using context and morphological features. These produce linguistically valid lemmas rather than stems and require annotated treebanks such as Universal Dependencies. They are relevant as a comparison point but should not be conflated with stemming, since their output and training requirements differ.

#### 6.5.4 Neural morphological segmentation

Statistical morphological segmentation tools such as Morfessor 2.0 (Smit et al., 2014) segment words into morpheme sequences rather than producing a single stem, and neural extensions of this approach have since been explored in the literature. Segmentation can serve as a preprocessing step for stem identification but is not equivalent to stemming, as it does not designate which segment is the stem.

#### 6.5.5 Multilingual transfer learning

Cross-lingual transfer from high-resource to related low-resource languages has shown promise for morphological tasks. Zhu et al. (2019) demonstrated that sub-word information transfer improves morphological task performance in genuinely low-resource languages, though gains depend heavily on the typological similarity between source and target languages.

#### 6.5.6 Subword tokenization as an implicit representation technique

Byte Pair Encoding, Unigram LM, and SentencePiece, as used in mBERT and XLM-RoBERTa, decompose words into frequency-based sub-word units. These units often approximate morpheme boundaries but are optimized for compression, not linguistic validity. Sub-word tokenization is therefore an implicit representation technique that can substitute for stemming in some downstream neural pipelines, but it does not perform explicit stemming and provides no guarantee of valid stems.

#### 6.5.7 Prompting and instruction-tuned LLMs for explicit stemming

Wang et al. (2024) evaluated whether instruction-tuned large language models such as GPT-3.5 can perform explicit stemming within information retrieval pipelines. Their findings were mixed: LLMs produced reasonable stems for high-resource languages but failed inconsistently on morphologically irregular and low-resource forms, and introduced latency and cost overheads that limit practical deployment. LLM-based stemming for truly resource-poor languages remains largely untested.

#### 6.5.8 Qualified assessment

It would be too categorical to claim that rule-based and hybrid methods are the only viable option for languages lacking annotated corpora. Several intermediate strategies exist and merit consideration: weak supervision using small seed lexicons; cross-lingual transfer from typologically related languages; multilingual pre-training that shares sub-word vocabularies; synthetic data augmentation for character-level models; finite-state morphological analysers, which encode linguistic knowledge without requiring large corpora; and community-created lexical resources, which can supply the dictionary component of hybrid stemmers. The more accurate conclusion is that rule-based and hybrid methods remain the most practical and interpretable choice for many resource-poor languages today, but that weakly supervised and transfer-based neural methods represent a promising and underexplored direction as multilingual resources continue to expand.

## 7 Comparison setup

In this survey paper, several base papers are seen through different parameters. Some of the parameters can be seen in the below table based on the list of the methods used to evaluate, similar approaches, comparison of the results of the proposed solution with similar approaches, and keywords used for comparison.

### 7.1 Structured evidence synthesis

Table 9 presents a structured evidence synthesis of representative primary stemming studies included in this review. Each entry records the language and language family, morphological characteristics, method category, task classification, dataset and size, evaluation design, metric reported, comparison baseline, principal result, and key limitation. This structure enables cross-study comparison that goes beyond individual paper summaries. To avoid over-representing Arabic, which accounts for the largest share of retrieved primary studies, Table 9 limits Arabic to a single representative study; the remaining Arabic studies are analyzed in detail in Section 5.1.1.

**Table 9:** Structured evidence synthesis of representative primary stemming studies included in this review.

Study	Language (family, profile)	Method	Dataset (size)	Result (metric vs. baseline)	Key limitation
Ali et al. (2017)	Urdu (Indo-Aryan, fusional)	Rule-based	Urdu news headlines (4 categories)	Higher accuracy than NB and SVM classifiers	Manual rules; limited scalability
Alnaied et al. (2020)	Arabic (Afro-Asiatic, root-based)	Rule-based	TREC 2002 AFP (~150K docs)	MAP 0.34 vs. LUCENE 0.27 and FARASA 0.28	Does not handle all broken plurals
Saharia et al. (2013)	Assamese, Bengali, Bodo, BM (Indo-Aryan, agglutinative)	Hybrid (HMM + rule)	Proprietary corpus (size not stated)	Higher accuracy than rule-only baseline; best on Assamese	No public dataset or code
Nathani et al. (2020)	Sindhi (Indo-Aryan, fusional)	Unsupervised (MDL)	Proprietary corpus (size not stated)	Effective stem discovery vs. rule-based baselines	UI and OI not quantified
Alotaibi and Gupta (2018)	English, Bengali, Marathi, Hungarian (multi)	Unsupervised	CLEF, TREC, FIRE (~100K docs)	Higher MAP than GRAS, Porter, YASS	Computationally expensive
Özbey and Karcili (2021)	Turkish (Turkic, agglutinative)	Unsupervised (SDDM/PDDM)	Turkish Wikipedia (363K words)	*O*(*M*) vs. *O*(*M*^2^) boundary detection in Morfessor 2.0	No downstream task evaluation
Singh and Gupta (2019)	English, Marathi, Bengali, Hungarian (multi)	Unsupervised	FIRE 2010, WSJ (~100K docs)	Higher MAP than Porter and co-occurrence stemmers	Misses language-specific morphology
Jabbar et al. (2019)	Urdu (Indo-Aryan, fusional)	Rule-based (UTS)	Urdu news corpus (size not stated)	Multi-stage pipeline improves over lightweight Urdu stemmer	No quantitative result reported
Gobin-Rahimbux et al. (2024)	Kreol Morisien (creole, partially rich)	Hybrid (KreolStem)	Proprietary corpus (size not stated)	Improved accuracy over rule-only baseline	Very limited language resources

Reported results state the metric and the comparison baseline; accuracy and F-measure denote intrinsic evaluation, while MAP and NMI denote extrinsic evaluation on retrieval or clustering tasks.

### 7.2 Cross-study synthesis

The evidence table reveals several patterns that are not visible from individual paper summaries. Why results differ across languages. Arabic stemming studies consistently report MAP gains of 0.06–0.13 over no-stemmer baselines when using rule-based methods on TREC collections. These gains are driven by Arabic's root-based morphology, where a single trilateral root underlies dozens of surface forms, making vocabulary reduction through stemming directly beneficial for retrieval. In contrast, agglutinative languages such as Turkish and Finnish show more modest and variable MAP improvements because their morpheme boundaries are more regular and sub-word tokenization already captures much of the relevant variation. For truly resource-poor languages such as Bishnupriya Manipuri and Sindhi, no standardized benchmark exists, making cross-study comparison impossible. Why metrics cannot be compared directly. Accuracy, F-measure, MAP, NMI, and clustering purity measure fundamentally different outcomes and must not be treated as equivalent. Accuracy measures the proportion of correctly stemmed words in an intrinsic evaluation against a gold standard. F-measure combines precision and recall of stem assignment. MAP measures ranked retrieval performance averaged across queries. NMI measures the mutual information between cluster assignments and gold labels, normalized for cluster size. Purity measures the fraction of dominant-class items in each cluster. A stemmer achieving 94% accuracy (Saharia et al., 2013) and a stemmer achieving MAP 0.34 (Alnaied et al., 2020) are not comparable because they measure different things on different tasks with different datasets. Table 10 maps each metric to its appropriate evaluation context. Table 10 maps each metric to its appropriate evaluation context and states which cross-metric comparisons are invalid.

**Table 10:** Mapping of evaluation metrics to their appropriate evaluation contexts, indicating which cross-metric comparisons are invalid.

Metric	Appropriate context	Inappropriate comparison
Accuracy	Intrinsic stemming correctness	Cannot be compared to MAP or F-measure
F-measure	Stem precision and recall balance	Cannot be compared to NMI or accuracy
MAP	Ranked information retrieval	Cannot be compared to clustering metrics
NMI	Document clustering quality	Cannot be compared to IR or intrinsic metrics
Purity	Cluster homogeneity	Cannot be compared to retrieval metrics
UI / OI	Stemming error characterization	Supplementary to any of the above

Availability of code and data. A critical limitation across the reviewed literature is the near-universal absence of publicly available code and datasets. Of the nine primary studies in Table 9, only studies using TREC 2002, FIRE 2010, and Universal Dependencies provide publicly accessible evaluation data. The majority of studies use proprietary or institution-specific corpora that cannot be obtained, making replication impossible. This is the single largest barrier to progress in stemming for resource-poor languages.

Table 11 provides a concise comparison of major stemming paradigms, highlighting their core principles, data requirements, and strengths.

**Table 11:** Comparative analysis of stemming paradigms and their suitability for resource-poor languages.

Paradigm	Core idea	Data need	Typical use	Key strength
Rule-based	Linguistic rules and affix stripping	Very low	Classical resource-poor languages	Interpretable; effective with minimal data
Statistical/unsupervised	Pattern induction and segmentation	Low–moderate	Morphological analysis	Scalable; low reliance on expert rules
Neural/transformer-based	Neural sequence and sub-word modeling	High	Morphology modeling, translation	Captures complex and contextual morphology
Hybrid	Rules with statistical/neural refinement	Moderate	Balanced morphological tasks	Better trade-off between data and rules
Segmentation-centric	Morphology-aware sub-word units	Moderate	Tokenization and segmentation	Improves morphological fidelity

The comparison which is shown above is based on the experimental behavior of all the algorithms. The below Table 12 presents the evaluation strategies, comparison baselines, and key performance metrics adopted in representative stemming studies. The effectiveness of stemming algorithms is commonly evaluated using a combination of classification-, clustering-, and retrieval-based metrics to capture both intrinsic accuracy and downstream task impact.

**Table 12:** Evaluation strategies and comparative baselines used in related stemming studies.

References	Evaluation methods	Comparison baselines	Key metrics/keywords
Ali et al. (2017)	Minimum word length, frequency counts, n-grams, HMM	Lightweight Urdu stemmer, short-text classifiers (NB, SVM)	Prefix/suffix rules, TP, FP
Alotaibi and Gupta (2018)	TERRIER IR system, IFB2 weighting model	GRAS, Porter, YASS	MAP, probabilistic divergence

Evaluation is either *intrinsic* (stem correctness: accuracy, F-measure, UI, and OI) or *extrinsic* (downstream impact: MAP, NMI, purity)—different units, not directly comparable. In Equations 1–10, TP/TN/FP/FN follow standard definitions; UF/OF are pairwise under-/over-stemming counts (Paice, 1994); *V*_*original*_/*V*_*stemmed*_ are pre-/post-stemming vocabulary sizes; *I*, *H* are mutual information and entropy for NMI. Macro- vs. micro-averaging across languages is inconsistently reported in the literature (Section 6.2).

The most widely used evaluation measures in the literature are defined as follows.


$$\begin{array}{l} \text{Precision}=\frac{TP}{TP+FP} \end{array}$$ (2)

$$\begin{array}{l} \text{Recall}=\frac{TP}{TP+FN} \end{array}$$ (3)

$$\begin{array}{l} \text{Accuracy}=\frac{TP+TN}{TP+TN+FP+FN} \end{array}$$ (4)

$$\begin{array}{l} \text{Under-stemming Index (UI)}=\frac{UF}{TP+UF} \end{array}$$ (5)

$$\begin{array}{l} \text{Over-stemming Index (OI)}=\frac{OF}{TP+OF} \end{array}$$ (6)

$$\begin{array}{l} \text{Vocabulary Reduction Ratio (VRR)}=\frac{\left|V_{\text{original}}|-|V_{\text{stemmed}}\right|}{\left|V_{\text{original}}\right|} \end{array}$$ (7)

$$\begin{array}{l} \text{NMI}=\frac{2\times I\left(X;Y\right)}{H\left(X\right)+H\left(Y\right)} \end{array}$$ (8)

$$\begin{array}{l} r=\frac{\sum \left(x_{i}-\bar{x}\right)\left(y_{i}-ȳ\right)}{\sqrt{\sum {\left(x_{i}-\bar{x}\right)}^{2}\sum {\left(y_{i}-ȳ\right)}^{2}}} \end{array}$$ (9)

$$\begin{array}{l} \text{MAP}=\frac{1}{Q}\overset{Q}{\underset{q=1}{\sum }}AP\left(q\right) \end{array}$$ (10)

Table 13 summarizes the strengths and limitations of major stemming paradigms for resource-poor languages.

**Table 13:** Consolidated advantages and limitations of major stemming paradigms for resource-poor languages, highlighting trade-offs in linguistic coverage, scalability, efficiency, and downstream task performance.

Method category (References)	Key advantages	Key limitations
Rule-based stemmers (Urdu, Arabic, Indonesian) (Ali et al., 2017; Jaafar et al., 2017; Alnaied et al., 2020; Alshalabi et al., 2022b; Rianto et al., 2021)	Interpretable linguistic rules; effective in truly low-resource settings; supports loanwords and core-letter identification; improves PoS tagging and IR accuracy	Manual rule design; limited scalability; higher execution time due to lexical validation; incomplete rule coverage
Unsupervised/statistical stemmers (MDL, co-occurrence, G2P) (Nathani et al., 2020; Suyanto et al., 2022; Özbey and Karcili, 2021; Singh and Gupta, 2019)	Language-agnostic; low reliance on expert rules; scalable across languages; reduced time complexity	Weaker performance for highly inflected forms; sensitive to segmentation quality; may underperform rule-based lemmatizers
Hybrid/Joint learning approaches (Bölücü and Can, 2019)	Joint modeling (PoS + stemming) improves accuracy over standalone stemmers; handles OOV words effectively	Moderate data requirements; performance varies across languages
Application-driven evaluations (IR, similarity, topic modeling) (Alhawarat et al., 2021; Ma et al., 2020)	Demonstrates downstream benefits in retrieval, semantic similarity, clustering, and summarization tasks	Results depend on weighting schemes, task setup, and evaluation metrics; limited generalization across tasks

## 8 Discussion

Stemming is a crucial step in natural language processing, particularly for languages with rich morphology. There are two main types of stemming algorithms that are discussed here: statistical and rule-based. The discussion section will be divided into two subsections, one for each type of algorithm.

### 8.1 Statistical stemming methods

Statistical stemming algorithms use probabilistic models to determine the base form of a word. These algorithms typically rely on corpus analysis and machine learning techniques to identify patterns and regularities in the data. Statistical stemming algorithms are particularly effective for languages with rich morphology, where words can take on many different inflections and forms. These algorithms can help to map different inflected forms of a word to a single base form, which can simplify text processing tasks and improve the accuracy of NLP (natural language processing) applications. One of the main benefits of statistical stemming is its ability to handle variations in word forms, including different tenses, plurals, and other inflections. This type of method can be useful for languages such as Sindhi, Turkish, Finnish, Hungarian, English, Turkish, Marathi, and Bengali (Ali et al., 2017; Alnaied et al., 2020; Jabbar et al., 2019; Alshalabi et al., 2022b). Moreover, statistical stemming algorithms can be trained using large amounts of textual data, that help to learn patterns and relationships between words and their various inflected forms. This training enhances the accuracy of algorithms, making them more effective in handling variations in language use. However, there are limitations associated with statistical approaches. One primary limitation is to handle irregular words and rare forms, which can lead to errors in the stemming process. This can result in the loss of important information, as the base form of a word may not fully convey the intended meaning of the original word. Another potential disadvantage is the computational complexity of stemming algorithms, particularly for large datasets or languages with complex morphology. This can result in longer processing times and increased resource usage, which may limit the scalability and practicality of statistical stemming approaches. In general, statistical stemming can greatly enhance the efficiency and accuracy of natural language processing applications, especially for languages that possess complex morphology. However, used languages should have enough resources, as statistical methods are not suitable for low-resource languages. For low-resource languages, rule-based stemming methods are preferable.

### 8.2 Rule-based stemming algorithm

The rule-based stemmer algorithms rely on a list of rules that determine how suffixes or prefixes can be removed from the target words in order to form their stems. This process is often employed in NLP to enhance the accuracy of language models, and other text analysis tools. Rule-based stemming is more effective for some languages than others, depending on the language's grammatical structure and morphological complexity. Rule-based is specially used for resource-poor languages. Rule-based stemming algorithms rely on manually constructed rules that are based on linguistic knowledge of the language, which can be developed by linguists or language experts without the need for large amounts of data. Rule-based stemming is more effective for languages with regular morphology and clear inflectional and derivational patterns, such as Urdu, Arabic, Finnish, Turkish, and Arabic (Porter, 1980; Alnaied et al., 2020). The advantage of rule-based stemming is the reduction in text corpus size and simplified text analysis by reducing the number of word forms. However, rule-based stemming can sometimes result in errors or incorrect results when the rules fail to account for all possible inflectional or derivational variations. Overall, rule-based stemming algorithms are the preferred choice for resource-poor languages due to their unavailability of enough linguistic knowledge and resources.

### 8.3 Hybrid stemmer

Hybrid stemmer combines machine learning-based approaches and rule-based approaches at the same time. In some situations, employing a Hybrid algorithm is a better option such as dealing with languages that have a moderate amount of resources available, but still require some level of linguistic expertise to accurately stem words. Languages that have a complex morphology, such as Arabic, Bengali, Bodo, and Bishnupriya Manipuri (Steinberger, 2012) are a better option to take a hybrid approach. In such languages, simply a rule-based approach may not capture all possible variations of the word. That is the reason employing machine learning-based methods requires applying rules to cut off the affixes used. Overall, hybrid algorithms are the best option for languages that have a moderate number of resources available but still require some level of linguistic expertise to accurately stem words.

The comparative analysis reveals that no single stemming paradigm universally outperforms others across languages and applications. Statistical and neural stemmers excel when sufficient data is available, while rule-based methods remain indispensable for truly low-resource settings (Steinberger, 2012). Hybrid approaches that integrate linguistic rules with probabilistic or neural models offer a balanced trade-off between accuracy and scalability. Future research should focus on adaptive, language-aware stemming frameworks capable of leveraging both symbolic and data-driven knowledge. In conclusion, stemming is a critical aspect of natural language processing and its many applications, such as information retrieval systems. This survey paper has provided an overview of different stemming algorithms used across various languages and applications. The algorithms are broadly classified into two categories: rule-based and statistical approaches. Statistical approaches are further classified into supervised and unsupervised algorithms. The paper discusses the advantages and disadvantages of each category, along with the major challenges faced during their implementation. Different types of stemming algorithms have also been compared across multiple languages, and the evaluation metrics used to analyze these algorithms have been discussed. In general, rule-based algorithms are more suitable when dealing with resource-poor languages, whereas for languages with sufficient linguistic and annotated resources, statistical approaches improve stemmer efficiency by handling irregular and unseen word forms. In certain scenarios, hybrid algorithms can be effectively employed, particularly for languages such as Arabic. The survey highlights that the choice of a stemming algorithm depends on specific application requirements as well as the morphological characteristics of the target language. It also emphasizes that a strong understanding of language morphology and structure plays a crucial role in effective stemming.

### 8.4 Open challenges and future research directions

Several critical challenges remain unresolved in stemming for resource-poor languages. First, there is no standardized evaluation benchmark for cross-linguistic stemming comparison. Each study uses different datasets, metrics, and baselines, making it impossible to objectively rank stemming approaches across language families. Second, over-stemming and under-stemming rates are rarely reported, despite being the most linguistically meaningful indicators of stemmer quality. Future work must adopt unified error reporting. Third, code-switched and transliterated text—increasingly common in social media for languages such as Urdu, Hindi, and Arabic—is entirely unaddressed by existing stemmers. None of the methods reviewed handles mixed-script or romanized input. Fourth, LLM-based stemming is promising but fundamentally untested for resource-poor languages. Whether models like GPT-4 or multilingual LLaMA can perform morphologically valid stemming for Bishnupriya Manipuri, Bodo, or Sindhi is an open empirical question. Fifth, ethical concerns around script normalization are absent from the literature. Aggressive stemming can erase morphological distinctions that carry grammatical gender, honorifics, or sociolinguistic meaning—particularly in South Asian and Semitic languages. This dimension requires attention as NLP tools are deployed in real communities.

## 9 Ethical considerations

Stemming, though a technical preprocessing step, carries sociolinguistic implications when applied to minority and endangered languages. Overly aggressive affix stripping can collapse morphological distinctions that encode grammatical gender, social register, or cultural meaning. For languages such as Bishnupriya Manipuri and Bodo, where annotated resources are scarce and community oversight of NLP tools is minimal, stemmer design decisions made without linguistic consultation risk encoding bias into downstream systems. Researchers developing stemmers for resource-poor languages should involve native speaker communities and document failure modes transparently.

## 10 Limitations of this review

While this review critically examines the limitations of stemming methods, the review process itself is subject to several constraints that should be acknowledged. Database and search-language restrictions. The literature search was conducted in five English-language databases using English search terms. Relevant studies published in other languages, or indexed only in regional databases, may not have been retrieved. This is a particular concern for research on resource-poor languages, which is sometimes published in national venues, local journals, or in the language of the community itself. Publication bias. The review draws on peer-reviewed published studies, which are more likely to report positive results. Stemming approaches that were attempted and performed poorly may be underrepresented in the retrieved literature, potentially inflating the apparent effectiveness of published methods. Exclusion of unavailable full texts. Studies whose full text could not be accessed were excluded at the screening stage. Since access barriers disproportionately affect publications from institutions in developing regions, this exclusion criterion may have systematically removed relevant work on exactly the languages this review targets. Unequal coverage of languages and families. As discussed in Section 2.4, the retrieved literature is heavily concentrated on Afro-Asiatic and Indo-Aryan languages. Conclusions drawn in this review are therefore most reliable for those families and should be generalized to Bantu, Dravidian, Austronesian, and other underrepresented families with caution. Heterogeneous tasks and metrics. The included studies evaluate stemming using different tasks, datasets, and metrics that are not directly comparable, as detailed in Section 7.2. This heterogeneity prevented any formal quantitative synthesis; a meta-analysis of stemmer performance across studies was not possible, and the comparisons presented here are necessarily qualitative. Reliance on reported results. All performance figures cited in this review are taken from the original publications as reported. None of the reviewed stemmers were independently re-implemented or re-evaluated by the authors of this review. Given the widespread unavailability of code and datasets noted in Section 7.2, independent verification of most reported results is currently infeasible. Possible omission of community-published resources. Lexicons, stemmers, and corpora created by language communities and published outside academic venues—for example in open-source repositories, community wikis, or language technology foundations—fall outside the scope of a database-driven academic search and are not systematically represented in this review, despite their practical importance for resource-poor languages.

## 11 Conclusion

This survey has presented a comprehensive and systematic review of stemming techniques for resource-poor and morphologically rich languages, covering rule-based, statistical, unsupervised, hybrid, and emerging neural-assisted paradigms across five major linguistic families: Afro-Asiatic, Indo-Aryan, Turkic, Uralic, and multilingual settings. The analysis confirms that rule-based stemmers remain the most practical choice for truly low-resource languages due to their minimal data requirements and interpretability, while hybrid and joint learning models offer improved accuracy where modest annotated resources exist. Statistical and neural approaches show strong potential but are constrained by data scarcity and computational demands in the languages this survey targets. Key open challenges include the absence of a unified evaluation benchmark, underreporting of stemming error indices, and the complete lack of solutions for code-switched and transliterated text. Future work should prioritize adaptive, language-aware stemming frameworks that combine symbolic linguistic knowledge with lightweight statistical learning, and that are designed in collaboration with the speaker communities of the languages they serve.
